# British and Japanese international retirement migration and creative responses to health and care challenges: a bricolage perspective

**DOI:** 10.1186/s40878-020-00217-x

**Published:** 2021-02-26

**Authors:** Kelly Hall, Mayumi Ono, Ayako Kohno

**Affiliations:** 1 University of Birmingham, Birmingham, UK; 2 Notre Dame Seishin University, Okayama, Japan; 3 Kyoto University, Kyoto, Japan

**Keywords:** International retirement migration, Spain, Malaysia, Bricolage, Care, Health, Transnational

## Abstract

Most research on international retirement migration has focused on the Western context and the motivations and lifestyle choices of migrants when they are healthy. This paper instead explores how British retirees in Spain and Japanese retirees in Malaysia respond to declining health and increasing care needs through bricolage as they begin to ‘age in place’. The paper combines qualitative interviews, focus groups and observations collected by the authors from 215 British and Japanese international retirement migrants. We focus on two key types of bricolage behaviour: ‘within-system bricolage’ undertaken by migrants to help them access and navigate existing health and care systems; and ‘added-to-system bricolage’ that is enacted to fill gaps in health and care provision. Our analysis suggests that IRMs engage in ‘transnational care bricolage’ by combining multiple economic, social and legal resources across local and transnational spaces to address their health and care needs.

## Introduction

Most international retirement migration (IRM) research has focused on the Western context and much of this has explored the motivations, lifestyle choices and social networks of ‘third-age’ migrants when they are healthy and mobile. This paper is the first to present a cross-cultural comparison of British and Japanese international retirement migrants (IRMs) living in Spain and Malaysia respectively, focusing on how they respond to health and care challenges as they ‘age in place’. We offer two novel contributions to the field of migration. First, we provide an original comparison of British and Japanese IRMs. Whilst seemingly different cultural and structural contexts, British retirees in Spain and Japanese retirees in Malaysia share many common characteristics and face similar challenges. Both groups of IRMs undertake ‘lifestyle’ migration as a means to a better quality of life in older age (Benson and O'Reilly 2009). Neither group fully integrate into the host society, but are embedded in migrant communities that provide socialisation, a sense of ethnic belonging and play an active support role (Oliver 2017). IRMs also maintain transnational ties with the homeland that can be used instrumentally for care and support (Hall and Hardill 2016; Ono 2018). The lives of these IRMs are therefore complex and often constructed over national borders. We draw on qualitative research in Spain and Malaysia to explore how local and transnational resources are crucial to support life when migrants age away from the more established support systems of the homeland.

Second, the study extends existing conceptualisations of bricolage to illustrate how British and Japanese IRMs mobilise and combine resources in response to health and care challenges. We also extend the growing body of research on migrant health, which suggests that migrants can lack appropriate and approachable healthcare resources and so ‘make do’ through bricolage (Phillimore et al. 2018; 2019). Most migrant health research has focused on ‘resource-poor’ migrants, but we argue that IRMs can face similar difficulties due to their legal status, financial capital, knowledge, trust, language and cultural barriers (Hall and Hardill 2016; Kohno et al. 2016b; Phillimore et al. 2019). Our research suggests that IRMs engage in ‘transnational care bricolage’ to access and ‘add to’ existing health and care provision by utilising and combining multiple resources within and across the local, national and transnational spaces within which they are embedded.

In the next part of the paper, we provide an overview of existing literature on IRM in Spain and Malaysia, and then consider bricolage theory in relation to IRM. We then explain our methodology and set out the findings that draws on qualitative data collected from 215 IRMs. Our findings show how IRMs engage in two key types of bricolage behaviour: ‘within-system bricolage’ undertaken by IRMs to help them access and navigate existing health and care systems; and ‘added-to-system bricolage’ that is enacted to fill gaps in health and care provision. Our analysis indicates the multidimensional nature of bricolage solutions that exist within and across local and transnational spaces using multiple economic, social and legal resources.

## Retiring to Spain and Malaysia

Associated with an ‘active’ ageing philosophy (Oliver 2008), IRM usually involves migration to warmer and cheaper countries that enable a more active, outdoor lifestyle than the one left behind (Benson and O'Reilly 2009). Often viewed as a ‘Western’ phenomenon e.g. from Northern to Southern Europe or North America to Central/South America, IRM has become more widespread, particularly in the Asian context. Growing numbers of Europeans are retiring in South-East Asia (Green 2014; Green 2015) and Malaysia has become a favoured retirement destination for the Japanese (Ono 2018; Ormond and Nah 2019; Toyota and Xiang 2012). IRM to and from Asian counties is a relatively new but rapidly growing phenomenon, and there are increasing numbers of Asian and Western older people using transnational mobility to seek (low-cost) care in South-East Asia (Horn et al. 2015; Ono 2015a; Ormond and Toyota 2016). Research has also begun to explore the growing vulnerabilities of IRMs in Europe or Asia as they age (Sampaio 2018; Ciobanu et al. 2017; Green 2014; Hall and Hardill 2016), although none has compared the two contexts in relation to health and care. In this paper, we therefore explore how IRMs in both Europe and Asia respond to health and care challenges as they age in place.

Spain has been the most popular destination for older British (and other Northern European) nationals for many decades because of the good climate, relatively low living costs, established British communities and good tourist infrastructure, especially in the Spanish coastal regions where most retirees reside (O’Reilly 2017). Around 117,000 British nationals receive their state pension in Spain (Benton 2017), but such estimates do not include those who are not legally resident or have retired before state pension age (Finch et al. 2010). Retirement migration to Spain accelerated with the creation of the EU and associated free movement principles in 1993 that enabled EU citizens to reside in EU member states and for British state pensioners to access free public healthcare to the same level as a Spanish citizen. Whilst language and cultural barriers do persist, IRMs report Spanish healthcare to be good and few use private healthcare services (Hall 2016). Public social care is underpinned by residency and so British nationals who have been legally resident in Spain for 5 years are entitled to support from Spanish Social Services (Calzada 2017). Whilst some British IRMs are affluent, EU citizenship means that some IRMs can and do move to Spain with only the small British state pension leaving them dependent on public welfare systems (Hall and Hardill 2016). The EU withdrawal agreement has guaranteed existing welfare rights for British IRMs who are legally resident in Spain, but it is yet unknown if the same rights will continue for new migrants once the UK fully withdraws from the EU in 2021.

Whilst regional integration has accelerated IRM in Europe, in Asia it is a relatively new phenomenon where national borders are much less permeable for retirement migration (Toyota et al. 2006). Japanese retirees began moving to Southeast Asia in the late 1990s with the issuing of special visas for foreign retirees (Yamashita 2012). Malaysia introduced the ‘Silver Hair Programme’ in 1988 (Chee 2007), which was restructured in 2002 into ‘Malaysia My Second Home’ (MM2H) that offers 10-year multiple entry visas, and the option to purchase property in Malaysia. Between 2002 and 2018, 4778 Japanese obtained MM2H visas, with the number of applicants rapidly increasing from 195 in 2010 to 423 in 2011 and 816 in 2012, an increase arguably triggered by the 2011 earthquake that caused massive devastation in Japan (Ministry of Tourism and Culture Malaysia 2020). Applicants to MM2H are required to have liquid assets worth RM350,000 (approx. £66,000), a monthly income of RM10,000 (£1800) and medical insurance (Ministry of Tourism and Culture Malaysia 2020). Malaysia is now the most desirable destination for Japanese retirees (Long Stay Foundation of Japan 2019), attracted by the possibility of a more financially sustainable lifestyle and new social and cultural experiences (Ono 2015b). Popular retirement destinations include Penang, the Cameron Highlands, and Ipoh, although the largest retirement community and most significant Japanese infrastructure is in Kuala Lumpur. In contrast to the British, the Japanese rely almost exclusively on private healthcare facilities via private medical insurance (Kohno et al. 2016a).

Neither British nor Japanese IRMs tend to speak the local language (Oliver 2017), and instead create ‘community belonging’ through local and transnational networks of reciprocal support centred around ethnically homogeneous retirement communities (Legido-Quigley and McKee 2012; Ono 2015a). Community is characterised by trust and reciprocity through friendships, voluntary activity and informal exchange (Hall and Hardill 2016; Haas 2013). It has been widely noted that IRMs maintain strong social, political and cultural ties to their homeland and so live transnational lifestyles that include ongoing relationships with friends and family ‘back home’ that they may utilise for care (Hieda et al. 2013; Ciobanu et al. 2017). Many IRMs choose to return to the homeland in later life, whilst others, especially British IRMs, choose or are forced to stay when they age and need care (Giner-Monfort et al. 2016). The intersection of old age and migration can bring particular challenges due to the transnational context within which IRMs are located that restricts access to family care and public health, care and welfare services (Gavanas 2017; Ahmed and Hall 2016; Ono 2018). We therefore argue that IRMs develop creative solutions to address health and care related challenges through bricolage.

## IRM and transnational care bricolage

The concept of bricolage, originally coined by Lévi-Strauss (Levi-Strauss 1966, p. 17), refers to ‘making do with what is at hand’ through the creative mobilisation and (re) combining of resources for new purposes or in response to new problems/opportunities. Resources can include material goods, people and legal frameworks (Baker and Nelson 2005). To date, most bricolage literature has focused on entrepreneurship (Hall et al. 2019; Baker and Nelson 2005) and assumes that resources ‘at hand’ are locally based and sedentary. More recently the concept has been applied to the field of migration (Phillimore et al. 2019) and research has explored how transnational and mobile resources are utilised for bricolage. Phillimore et al. (2018, 2019) coined the term ‘healthcare bricolage’ to understand how migrants in superdiverse neighbourhoods engage in the ‘creative mobilisation, use and re-use, of wide ranging resources, including multiple knowledges, ideas, materials and networks in order to address particular health concerns’ (Phillimore et al. 2018, p. 6). We extend this analysis to suggest that the ways in which migrants connect resources from across localities, the world, and different medical and care systems can be conceptualised as ‘transnational care bricolage’. In our paper, we focus on two forms of bricolage; ‘within-system-bricolage’ undertaken by migrants to help them access and navigate existing health and care systems e.g. by translating healthcare information; and ‘added-to-system bricolage’ that is enacted to supplement or ‘add-to’ existing health and care provision e.g. ‘out-of-pocket’ or voluntary services (Phillimore et al. 2019).

Prior research has explored how IRMs navigate legal and political structures to access to formal health and social care systems (La Parra and Mateo 2008; Calzada 2017; Gehring 2017), and how IRMs create their own informal community based care and support services (Haas 2013; Toyota and Xiang 2012). IRMs may also seek care from families across national borders through what has become known as ‘transnational care’ (Baldassar 2014; Kilkey and Merla 2014). However, this body of research often fails to recognise the complex interplay of legal structures, economic resources, and family, community and other social ties that may be utilised by migrants in response to health and care challenges. This is especially the case in migrant communities, where existing research fails to acknowledge health and care as an ecosystem combining multiple services, sources of information, and networks that may be accessed locally, translocally or transnationally (Phillimore et al. 2018). We therefore employ a bricolage perspective to help us understand the creative strategies employed by IRMs to combine and connect social, economic and legal resources within and across national borders to address health and care challenges.

## Methodology

The paper draws on qualitative data from three studies undertaken separately by the authors between 2006 and 2019. Each of the studies explored the experiences of ageing for IRMs, focusing on how migrants accessed health, care and other support systems in the country of migration and transnationally. All three studies adopted a similar narrative approach and through the combination of individual in-depth interviews, focus groups and observations, we were able to understand the individual and collective life stories of participants. Central to all of our research was the importance of the personal experiences of our participants, and so we each encouraged them to tell their stories (Murphy and Dingwall 2003). This approach has been successfully adopted in studies of chronic illness and seeks to enable people to discuss their experience of illness/care, as well as the impact of illness on their social roles (Gilbert 2008). The qualitative approach employed in each of the studies therefore sought to understand the personal and subjective experiences of and interpretations of the social world, and how social interactions are embedded in the daily strategies and practices of everyday life (Lawler 2002).

All of the 215 participants in our studies were retired migrants aged between 51 and 95 living for all or most of the year in Malaysia or Spain. All of the younger participants had retired early for health reasons and/or were the spouse/partner of a retiree. Most of our participants lived in areas that had relatively large concentrations of IRMs: the Costa del Sol in Spain and Kuala Lumpur in Malaysia. The characteristics of participants in each of the three studies are set out in Table 1.

**Table 1:** Summary of Participants

	British	Japanese (Ono’s study)	Japanese (Kohno’s study)
**Participants**	68	117	30
**Gender**	42 female, 26 male	56 female, 61 male	16 female, 14 male
**Age, mean years**	75	65	65
**Age, range**	51–95	51–83	54–79
**Area of residence**	Costa del Sol	Kuala Lumpur (87), Cameron Highlands (13), Penang (13) Ipoh (4),	Kuala Lumpur (22), Ipoh (8)
**Research Period**	2017–2019	2006–2018	2015
**Years living abroad**	1.5–27	0.25–20	0.5–20
**Marital Status**	38 married, 3 co-habiting, 17 widowed, 5 single, 4 divorced, 1 unknown	103 married, 6 widowed, 8 single	26 married, 1 widowed, 3 single

Participants were mostly recruited through social and voluntary organisations in Malaysia and Spain, plus snowball sampling. All studies were undertaken with the welfare of participants in mind, and pseudonyms were used to protect the identity of participants. Ethical approval for each study was obtained as required through each of the host universities. All of the interviews were recorded and transcribed. The interviews in Malaysia were undertaken in Japanese and translated into English for analysis. Our research involved undertaking a secondary narrative analysis of the gathered data (Elliot et al. 2015; Heaton 1998), which is now a widely recognised methodology involving an in-depth examination of a theme or subset of prior data for the purpose of extending the primary work (Thorne 1994; Ahmed and Hall 2016). Each author had previously published from their own data, and so the data was combined into one new dataset and (re) analysed collectively to offer a crucial and original cross-cultural comparison of British and Japanese IRMs, which also extends our theoretical understanding of bricolage within a migration context.

**Fig. 1:**
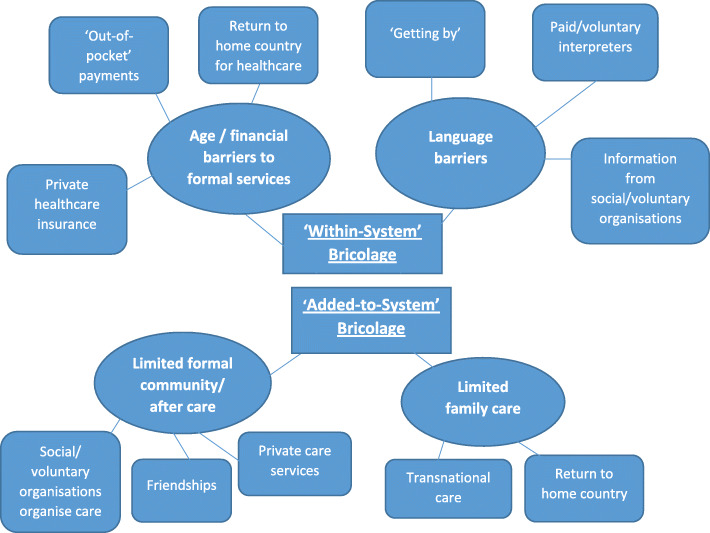
Thematic Coding Framework

In (re) analysing the qualitative data, we took a thematic approach. Whist we had no standardised data collection format or common interview guides, our joint analysis began with the development of a common coding framework (Attride-Stirling 2001) based on the theoretical interests and aims of this paper, centred around health/care related challenges and support strategies. Our coding framework was designed to enable a cross-country comparative analysis that identified the similarities and differences within and between the two IRM groups. Figure 1 presents the resulting thematic coding framework developed through our analysis, in which we identify the key health/care challenges encountered by participants, and the main strategies enacted to respond to these challenges.

A limitation associated with re-analysing existing data is a ‘lack of fit’ between old data and new research questions (Hammersley 2010), but we found that each of our studies had a shared focus on IRM health/care challenges that was sufficient to enable us to answer our research questions. A further limitation is insufficient contextual/cultural knowledge of the research setting (Hammersley 2010) so each team member first coded their own data independently using the coding framework. The two Japanese studies were then combined and analysed collectively, and then compared with the British data within each code. Our resulting thematic framework (Fig. 1) was developed from the analysis of qualitative data from our 215 respondents. The themes centred on the most commonly reported challenges and support strategies, and illustrative accounts of each emergent theme are presented in the findings that follow.

## Findings

The IRMs in our studies were found to engage in two key types of bricolage behaviour that are explained in the findings that follow. First, ‘within-system bricolage’ enacted by IRMs to help them access and navigate existing formal health and care systems within the public and/or private sectors. Second, ‘added-to-system bricolage’ that was supplementary to and filled gaps in health and care provision that was otherwise unavailable through formal routes (Phillimore et al. 2019). Our analysis include the challenges that led our respondents to bricolage, and indicates the multidimensional nature of bricolage solutions within and across local and transnational spaces using multiple economic, social and legal resources.

### ‘Within-system’ bricolage

For British and Japanese nationals, public healthcare systems are the normative approach to addressing a healthcare need; however, in Spain and Malaysia, public healthcare systems were not universally available and accessible to the IRMs. The retired British migrants in Spain (‘British’ from here onwards) who received a state pension were entitled to free public healthcare in Spain via EU citizenship rights. Whilst retired Japanese migrants in Malaysia (‘Japanese’ from here onwards) are entitled to use public healthcare services (for a fee), quality concerns led them to elect for private healthcare funded primarily through private insurance (Kohno et al. 2016b). Both groups of IRMs encountered challenges in accessing and/or navigating these ‘formal’ healthcare services and so responded through ‘within system’ bricolage activities involving the creative mobilisation of resources to make these services more accessible.

A key challenge for all of our participants was language barriers when seeking health/care related information and in medical appointments, as few spoke the local language. Language barriers were dealt with in a multitude of ways, including ‘getting by’ using hand gestures, personal translation tools (e.g. google translate) and writing down symptoms before appointments. However, such improvisation only got them so far and subsequently IRMs employed interpreters, or where these were too expensive, utilised free local services. For the British, a crucial resource were voluntary organisations, including a volunteer interpreter service that operated in Costa del Sol hospitals run by Spanish-speaking (mostly British) volunteers. The British also utilised other British-run voluntary organisations (e.g. Age Concern Espa*ñ*a, the Royal British Legion) for translation, but also for transport to hospital appointments, as Anne explained:

*[Volunteer] will be picking me up and taking me to the hospital … They take me to [and translate at] all my hospital appointments*. (Anne, 74, British)

In Malaysia, many of the Japanese spoke English but they did not speak Malaysian and so often had to use interpreters in medical settings. Interpreter services were often included in private hospital charges, but for the Japanese the main challenge was navigating private healthcare insurance systems. Unlike Spain, which has a plethora of British-run voluntary organisations, Malaysia has few voluntary organisations to support Japanese IRMs. Instead, the Japanese turned to their wider social networks for advice and information, including other IRMs and younger Japanese expatriates who were working as doctors, nurses, care workers and translators. Through these relationships, they were able to access health and care related advice, and obtain help to arrange medical insurance, book medical appointments and access care facilities. Ongoing personal relationships with MM2H agents, who had supported the Japanese when they first moved, were a crucial source of health and care advice, and provided translation at and transport to hospital. As Reika explained:

*The visa agent helps us to do everything we need, taking us to find a condominium and going to the hospital*. (Reika, 66, Japanese)

Social clubs, most notably the ‘Japan Club of Kuala Lumpur’ and the ‘Second Home Club’, also acted as a safety net for newly arrived Japanese IRMs, with our observations showing that they particularly support enrolment in medical insurance, and provide ongoing health and care related information. A ‘health support line’ was also established by a Japanese IRM, which provides Japanese language information on health and care services in Malaysia:

*When my husband became sick, the ‘[health support line]’ I am a member of, I call there and ask which hospital has a urology department. This system, it helped me a lot.* (Masako, 70s, Japanese, Focus Group)

Another key challenge for some participants was an inability to access formal (public or private) healthcare services due to age and/or financial barriers. Some of the Japanese found that when their health insurance policy lapsed post-migration, their deteriorating health and age led to increased premiums and so they were unable to afford to continue their cover. Similarly, a small number of the British were unable to access public healthcare because they were under state pension age and/or were not legally resident in Spain. Some of the British purchased private healthcare insurance, but in a similar scenario to the Japanese, pre-existing medical conditions and age meant costs were often unaffordable. In this scenario, the British and Japanese elected to make ‘out-of-pocket’ cash payments for private healthcare services. This option resulted in considerable cost, anxiety and led to the IRMs limiting their use of healthcare services. In a focus group, Hiroshi (73, Japanese) who was unable to renew his healthcare insurance due to the substantial cost explained, *“I was always very wary of how much they were going to bill us. We will try not to go to hospital as much as we can”*. Julia (53, British) is under state pension age and does not work so is not entitled to public healthcare. She stated ‘*I’ve got no health cover … [so] we try not to get ill*’.

Some of these IRMs responded by creatively utilising their transnational citizenship rights to access public healthcare provision from the home country. Legal frameworks are a recognised bricolage resource (Baker and Nelson 2005) and for the British, this involved retaining UK residency and returning to the UK for any planned healthcare treatments whilst using the European Health Insurance Card (EHIC) in Spain (to access free emergency healthcare). Retaining UK residency involved keeping an address in the UK at their own property or using a son/daughter/friend’s address. Julia explained that when in Spain, she uses her EHIC card and a ‘*pay-as-you go doctor’* costing ‘*150 euros a year’.* She also explained that she had retained an address in the UK at her father’s house to access the British NHS:

*I do need [medical treatment] at the moment, so I’m going to have to try and prearrange it through my GP in England...via my fathers.* (Julia, 53, British)

However, she goes on to recognise that travelling back and forth is only possible whilst she is healthy, and that her low income means this is not an option in the long-term. Similarly, some of the Japanese retained an address in Japan so that they could continue to use the Japanese health insurance system. To enable ongoing residency in Japan, a small number of participants were living in Malaysia as tourists rather than through MM2H, and so moved back and forth, in other words did ‘*visa runs*’ every 90 days:

*I did not even apply for MM2H and continue visa runs and stay in Malaysia for maximum 270 days a year. I registered my Malay host family’s address to the embassy. When I go to the hospital, I use my [credit] card.* (Tetsuo, 63, Japanese)

These examples of bricolage show how IRMs utilise and combine their legal and citizenship rights to access healthcare in the home and host countries. As prior research notes, IRMs can and do exploit the structural and legal gaps of their transnational lives by ‘picking and choosing’ the optimum healthcare provision available to them (Ackers and Dwyer 2002; Oliver 2017). This is a key example of ‘transnational care bricolage’ that involves the utilising and combining of legal, social and economic resources across national borders to access public healthcare. Some of the British who were legally resident in Spain also used their British citizenship rights to apply for exportable UK disability benefits, including Attendance Allowance. Some found that applying for the benefit from Spain a challenging process and so obtained help from a British voluntary organisation to complete the paperwork.

Whilst some of the IRMs retained residency in and returned temporarily to the home country to access healthcare, others spoke about return migration as a more permanent solution to their health and care challenges. Returning to access the more developed welfare system of the home country is a common strategy enacted by IRMs to address health and care needs in later life or if a health crisis arises (Giner-Monfort et al. 2016). Returning was a more common strategy for the Japanese than the British, which may be partly explained by the British having access to free healthcare in Spain, whilst the Japanese were likely to face increasing (private) healthcare costs as they aged. Furthermore, whilst the UK has a means-tested social care system (Thorlby et al. 2018), the introduction of long-term care insurance in Japan in 2000 led to almost universal care for those over 65 and may influence the decisions made by the Japanese to return once care needs arise, as Hanako explained:

*My husband is 75. I have to persuade him to return to Japan in the next few years for the time when we need care.* (Hanako, 68, Japanese)

These examples of within-system bricolage involve the combination of multiple resources to access formal healthcare services from the home and host countries. These same resources can however also restrict bricolage, particularly transnationally, with for example frequent travel and the retaining of an address in the home country being dependent on IRMs having sufficient economic, health and social resources in the first place.

### ‘Added-to-system’ bricolage

As the previous section highlighted, IRMs engaged in bricolage activities to access and navigate existing formal healthcare services. However, we also identified some health and care needs that could not be filled through existing service provision. Whilst the majority of IRMs in our studies referred positively to medical services, participants found that there was little or no community based care, including hospital aftercare, district nursing services, palliative care and domiciliary care (e.g. personal care such as help with washing/dressing). In Malaysia and Spain there is a cultural expectation that family members provide reablement and long-term care (León 2010; Samsudin et al. 2019), and very few IRMs have family living nearby (except a spouse who was often themselves elderly so unable to provide care). In Spain, Social Services are more developed than in Malaysia, but in both countries services are limited and patchy due to familial expectations and language/cultural barriers that restrict access where they do exist. Subsequently, no IRMs in our studies had accessed public community-based care. Participants found that they were discharged from hospital as soon as they were ‘medically well’ and as British retiree Geoff (aged 85), who had recently been in hospital explained, ‘*the operation was fine … the aftercare was virtually nil*’. IRMs therefore found they had to develop ‘added-to-system’ bricolage (Phillimore et al. 2019) solutions outside of formal care systems to address their needs.

At a local level, there is a strong sense of community among IRMs in Spain and Malaysia, characterised by friendships, social clubs and reciprocal exchange (Hall and Hardill 2016; Ono 2015a). This community was utilised as a resource to fill care gaps. Social clubs in Malaysia and voluntary organisations in Spain helped IRMs to organise and even provided informal community-based support. Judy explained how, when her husband was discharged from hospital late one night, she phoned a voluntary organisation for help:

*It was about one o’clock then they released him, and I was like “Crumbs, what am I going to do”? So I actually rang the [voluntary organization], and they sent somebody down for me … it’s an extended family to us*. (Judy, 73, British)

Friendships with other IRMs were also utilised for help and care in the home. Ruby, a British IRM (79, widowed) explained that in the absence of any aftercare, a friend had lived with her for 2 weeks after being discharged from hospital following a hip operation. Her friends also provided practical help in the home on a more ad-hoc basis (e.g. shopping, food preparation). Support between friends was multi-directional and inclusive, for example Ruby went on to explain how she used a ‘buddy system’ established by a voluntary organisation where small groups of IRMs who live alone, call each other every morning and evening to ‘check-in’. As Ruby explained, it was designed to ensure *“you made it through the night or day because sometimes you can go the whole day and not speak to anybody”.* These locally based ‘community-making’ bricolage practices often operated as mutual support networks based on reciprocity and offered a safety net by bringing together multiple local resources to bridge, fill and add to gaps in formal care and support (Olsson and O’Reilly 2017). In Malaysia, Japanese IRMs established the voluntary organisation *Otasuke Man Club,* that provided mutual assistance, particularly for new arrivals (Ono 2018; 2015a). Mitsuyo (60, Japanese), a founding member, explained, “*everyone starts by being helped and later on they will help other retirees*.”

Social networks were typically centred around the IRMs national/ethnic community, so whilst they helped to maintain a sense of national and cultural identity, it has been argued they may also serve to limit integration into the wider society (Olsson and O’Reilly 2017; Oliver 2017; Hieda et al. 2013). The majority of the IRMs in our studies therefore spent much of their time with other Japanese/British people, but this did not mean there was no local integration. The British referred to Spanish and Scandinavian friends and the Japanese spoke about friendships with Malaysians. Such cross-cultural exchange was more common among the Japanese, and included cross-generational and reciprocal relationships with younger Malaysian families. Saori explained that her local friends acted like family and offered care and support during times of need:

*We feel that a couple [Chinese Malaysian wife and Philippino husband] are like our daughter and son in Malaysia. They take care of us much more than our real children.* (Saori, 63, Japanese)

Saori’s experience highlights the importance of proximate support networks, especially during times of crisis when family living at a distance cannot be there. She did also maintain strong ties with her four children in Japan and many of the other IRMs referred to the emotional, practical and even financial help they received from children either virtually using video conferencing (e.g. Skype, FaceTime) and social media or through occasional visits. However, Saori’s experience and also prior research suggests that virtual support and care between children and their ageing parents is not a direct substitute for proximate care (Baldassar 2014; Kilkey and Merla 2014). Subsequently, some Japanese and British participants planned to return to live with children in the home country. For example, Sandra (80, British) explained how she was building an annex to her daughter’s house in the UK to which could return if her husband dies. Similarly, Eriko explained that she does not want to stay in Malaysia on her own after her husband dies and so plans to return to her daughter in Japan:

*We will be staying in Malaysia for the time being, but when my husband dies, I am not going to stay alone in Malaysia. I will go back to Japan, to my daughter’s place.* (Eriko, 70, Japanese)

Alternatively, those without close family relationships or who did not want to return often combined local and transnational resources to access care and support. Harriet explained that whilst her son wanted her to move back to the UK, she felt that Spain was her home. Therefore, her son visited from the UK when she came out of hospital, but when he left, he helped her to find locally based care:

*When I had the first operation, I needed a bit of care and help, and my son came over, and he said “We’ve got to get you some help when I go back. You can’t walk yet … you’ve got to have a bit of help”* (Harriet, 79, British)

Harriet and her son found that neither public Social Services nor informal (unpaid) local care solutions were sufficient to fully meet her needs and they had to turn to formal (paid) care services. Private Spanish care services were unfeasible due to language barriers (i.e. care staff rarely speak any English) and so they turned to the British community.

Within the IRM communities in Spain and Malaysia, private care markets have emerged that operate outside of existing statutory and local private provision and cater specifically to the needs of IRMs. In Spain, British people have set up residential/nursing homes and domiciliary care services with (mostly) British staff. Harriet started using a British-run care company who now visit every morning to help her get dressed/washed, and also take her to the hospital. Similarly, in Malaysia, private care facilities that cater to the needs of older Japanese retirees have been established, although in contrast to the British have involved cross-national collaborations between the Japanese and Malaysian communities. For example, a Malaysian GP established a nursing home for Japanese IRMs, where younger Japanese expatriates in Malaysia worked as staff and Japanese retirees helped as volunteers. The nursing home met the care needs of Japanese migrants for a short period, but in the long-term there was insufficient demand for the nursing home and it closed down. This indicates the fragility and fluidity of IRM communities (Oliver 2017) with bricolage often being for the purpose of ‘making-do’ in the short-term and in response to challenges as they arise (Phillimore et al. 2019).

Such private care services can be very expensive and many of our participants did not have the financial resources to pay for long-term care. Therefore, low-cost care arrangements have emerged within the IRM communities. For example, in Spain, Vera (80, British) cares for her husband with Alzheimer’s and explained that she was unable to access any care from Spanish Social Services due to long waiting lists and language barriers. She pays a British care company to help her for a few hours per week, but she needed respite care when she returned to the UK (her husband’s health means he cannot travel) and the high cost of paying the care company for 24/7 care led to her asking her British friends if they knew anyone that could help. A ‘friend of a friend’ suggested another British person who, for a small fee, stayed with her husband whilst she was away. Similarly, observations of the Second Home Club in in Japan found Toshiki (65) asked volunteers and other IRMs to help him arrange a Filipino domestic care worker to provide care for his elderly mother who lived with him in Malaysia. This creative combination of resources therefore allowed Vera and Toshiki to address their multiple care needs outside of any formal care systems.

These examples indicate the importance of community for IRMs as they navigate across and between formal and informal health and care services. We found examples of the informal and formal working in tandem, with voluntary organisations in Spain working closely with British care services and in some cases even paying the care costs for those on low incomes. In Malaysia, the Japanese IRM community was often used as a platform to launch and promote support services again indicating the intersection of the formal and informal health and care sectors.

## Discussion

Our findings illustrate that despite key structural differences in how British and Japanese IRMs access health and care services, they face many of the same language, cultural, legal and economic challenges that can restrict access to existing services and lead to gaps in provision. We extend Phillimore et al.’s (2018) notion of ‘healthcare bricolage’ and posit that whilst IRMs are more financially privileged than some other migrants, they can face similar barriers when it comes to accessing health, care and welfare services. They also engage in similar practices of bricolage and community-making that we term ‘transnational care bricolage’ involving the mobilisation and combination of multiple economic (e.g. assets), social (e.g. friendships) and legal (e.g. citizenship rights) resources within and across local and transnational spaces to address health and care challenges.

At a local level, we found that the British and Japanese IRM communities were instrumental in supporting participants to address their health and care needs. Practices of local bricolage involved micro level ‘community-making’ within, across and outside of existing structures (Olsson and O’Reilly 2017) and was important in both providing day-to-day care/support and mediating solutions to care deficits (Oliver 2017). These community solutions involved IRMs drawing on multiple resources and networks including friends, voluntary organisations and social clubs for help to access formal health services (within-system bricolage) and for help with community-based care that was not available through formal routes (added-to-system bricolage). Across all of our participants, bricolage involved combinations of individual and community solutions; for example, when Ruby came out of hospital her British friend looked after her at home, but when her friend had to leave, she signed up to the ‘buddy system’ of a voluntary organisation. Social and voluntary organisations often worked with families by for example helping worried children ‘back home’ find care for their elderly parents. These organisations also worked alongside statutory and private care/welfare services to assist IRMs (Oliver 2017) e.g. the ‘health support line’ in Malaysia, established by a Japanese IRM, was designed to help other Japanese IRMs navigate formal health services. Bricolage was therefore often an interactive process created and mediated by a broader community rather than the preserve of a single individual (Baker and Nelson 2005). One key difference between the two IRM groups was however the cross-cultural nature of many Japanese relationships at an individual and community level. The Japanese asked Malaysian friends for help and community services were often established by both Japanese and Malaysian people. The British were instead most likely to turn to British friends or volunteers, and there was less collaboration between the British and Spanish communities.

Bricolage theory has tended to assume the localisation of resources (Baker and Nelson 2005). ‘Local bricolage’, which is the (re) configuration of local services, networks and knowledge (e.g. the above combination of friendships, voluntary activity etc.) can be crucial to support IRMs, but we also argue that creating solutions through bricolage can require a wider set of resources than the localised (Cheung and Kwong 2017). IRMs therefore undertake ‘transnational care bricolage’ involving the combining of resources both within and across national borders to address health and care challenges. For some of our participants, transnational care bricolage involved mobilising multiple economic, social and legal resources (e.g. family, property, legal rights) to return to the homeland in old age, which is a widely recognised solution to address health and care challenges (Giner-Monfort et al. 2016; Kohno et al. 2016a). The welfare state of the home country therefore operated as a safety-net or what Olsson and O’Reilly (2017) refer to as a ‘security blanket’ - somewhere one can return to if all else fails. Others chose to remain in Spain or Malaysia to ‘age in place’, but utilised their transnational resources to access public health services. The stories of Julia and Tetsuo demonstrate how IRMs can use a combination of local (pay-as-you-go doctor) and transnational (address in the home country) resources to bricolage and access formal healthcare systems in both the home and host countries. Transnational bricolage has to date only been considered from a cultural dimension, including in the field of fashion, where it is described as ‘patchy and uneven’ combining mainstream and shadow economies (Mackie 2009). Similar uneven and patchy bricolage can also be seen with IRMs who often combine formal health and care resources with informal and unregulated community solutions. These examples demonstrate the way in which the state and community relations are not always aligned and migrants feel forced to pick and choose from various legal, social and economic resources to create the most effective health and care arrangements. These spaces that cut across the local and transnational could be referred to as what McKay (2016) calls ‘global shatter boxes’ i.e. spaces that exist within and beyond national borders, and where community-based care and support networks are more significant for wellbeing than any formal government bonds. Within shatter boxes, migrants forge and sustain care relations on the periphery of mainstream culture, relying instead on their own support systems, combining the informal economy and community, whilst at the same time forging cross-national ties.

The ability of IRMs to bricolage was of course dependent on the economic, social and legal resources they had at their disposal. Many IRMs had substantive networks of family, friends, social and voluntary organisations in the home and host countries that not only provided a sense of community and belonging, but were utilised as social capital (Casado-Diaz 2009) to access support, information and care. Others with limited social networks found themselves isolated and often had to cope on their own. Access to economic resources also varied considerably within and across the IRM groups. We found the Japanese overall had more financial capital than the British, which may be connected to the nature of the MM2H visa programme that includes financial and health insurance stipulations, meaning that only those with the financial capability can migrate. This is an example of what Gehring (2017) terms ‘legal gates’ that can limit and regulate the movement of people, and for the Japanese, mobility rights were connected to financial eligibility. Alternatively, such ‘legal gates’ did not exist for the British as EU principles and exportable social security rights enabled freedom of movement without any financial restrictions (at the time of interview). As such, we found considerable socio-economic diversity among the British IRMs (also see Hall and Hardill 2016), with some having only the basic state pension and so were dependent on state welfare, whilst others with substantial private pensions often owned property in the UK and were able to afford private health and care services. Such socio-economic diversity impacted on their ability to bricolage, so for example, an IRM that has no family in the home country and limited financial resources would be much less able to engage in transnational bricolage than an IRM with strong family ties and property in both countries.

The comparative nature of our paper offers some crucial insights into two IRM communities and the strategies that they use to access health, care and support. Japanese and British IRMs experience similar challenges associated with ageing away from the established welfare systems of the home country, however, citizenship and legal status is an important structural context for IRM and can create differences between British and Japanese IRM experiences. EU citizenship has permitted freedom of movement for British IRMs and grants their right to permanent residency with the premise of health and welfare coverage, enabling more British retirees to remain in Spain into their old age. This security has also enabled British IRMs in Spain, many of whom have been there for many decades, to establish large communities of support that have embedded and made possible bricolage practices. There are many social and voluntary organisations within the British community in Spain, many of which were set up by British nationals a number of decades ago and are both sustained by and operate to support a sizeable community of ageing British people. EU citizenship therefore provides basic health and welfare needs and stabilises British retirees aging in place.

Japanese IRMs, on the other hand, do not have formal citizenship status in Malaysia. They instead utilise financial eligibility to obtain ‘flexible citizenship’ (Ong 1999) and those that do not meet the financial criteria are inhibited from becoming IRMs in the first place. The Japanese IRM community in Malaysia also developed much later, from the mid-2000s, and is smaller than the British IRM community, so its function as a provider of health and care support is less established. Our data, collected over a 13-year period in Malaysia, showed that health and care services have become more accessible for the Japanese over time, with an expansion in private and voluntary services that both provide and facilitate access to health and care services (e.g. by providing Japanese language information). However, compared to the British, the Japanese IRM community is smaller, more fluid and so less stable, as the need to pay for healthcare means that retirement migration can only be sustained in the short-term until healthcare premiums become unaffordable. Out-of-pocket payments for healthcare are much higher, which can create financial insecurity and increases anxiety. Consequently, many Japanese IRMs may not be able to sustain their lives in Malaysia into old age, especially as dependence sets in and healthcare costs rise. This in turn creates a less stable Japanese community, and as our findings indicate, support services change rapidly as migrants come and go. Japanese are therefore more likely to seek cross-generational and cross-cultural support, maximizing the local resources that their informal networks as well as commercial services can offer. We may see similar patterns among the British IRM community in Spain over the coming years as a result of Brexit which has created considerable uncertainty and anxiety, especially in relation to the ongoing exportability of welfare rights (Hall et al. 2020). We can also refer to the impact of other structural changes, including the 2011 earthquake in Japan that triggered a rapid increase in emigration, and the more recent COVID-19 pandemic that may impact on the security of Japanese and British IRMs as they make decisions around whether to age in place or return to the home country.

## Conclusion

This paper presents an original cross-cultural comparison of British and Japanese IRMs and develops the concept of ‘transnational care bricolage’ to understand how IRM communities respond to health and care challenges by establishing solutions that mobilise, connect and combine economic, social and legal resources across local and transnational spaces. We demonstrate how transnational care bricolage is used to navigate formal health and care services (within-system), as well as supplement such provision and address needs that the formal system cannot meet (added-to-system). This comparative study reveals that health and care bricolage practices cut across local and transnational, formal and informal spaces and shape individual IRMs’ life strategies and practices of mobility/immobility. Our research highlights how the often fragile social, economic and political circumstances within which IRMs are often embedded, can impact on their micro level resources and force them to engage in ever more creative bricolage practices to address their needs. Alongside prior research (Hall and Hardill 2016; Ormond and Toyota 2016), we have uncovered considerable socio-economic diversity across and within IRM communities. British IRMs (and other Northern-European migrants more widely) arguably have a more privileged citizenship status as EU citizens and so are able to view migration as a self-realisation project in a way that is not always available to other migrants around the world (Olsson and O’Reilly (2017), including Japanese IRMs.

Our research has a number of practical benefits. It can help enable policy makers and practitioners to better understand how IRMs respond to health and care challenges and allow them to work with communities within and across national boundaries to ensure that IRM needs are met. We also offer an alternative interpretation of bricolage for researchers across social policy, gerontology and migration studies seeking to understand how ageing migrants draw on multiple local and transnational resources to respond to the challenges they face. With increasing numbers of IRMs settling in more disparate destinations around the globe, further comparative research is recommended to understand how different cultural contexts and welfare systems impact on older migrant experiences. For British IRMs, Brexit may lead to a scenario where many of the residency and welfare rights currently afforded to them are removed, thereby drawing retirees to alternative destinations, like Southeast Asia (Green 2014), where they will live alongside and under the same citizenship conditions as Japanese IRMs. Further research, undertaken post-Brexit, is needed to better understand IRM patterns and experiences in this new social and political context.

## Data Availability

All the datasets generated and analysed during the current study are not publically available due to participant confidentiality, but may be available from the corresponding author on reasonable request.
